# Opium and Opium Appetite

**Published:** 1871-09

**Authors:** 


*Opium and the Opium Appetite.* By ALONZO CALKINS, M.D.

The author of this work has given the profession, and especially the public the entire history of Opium; its commerce, statistics, pharmacology, physiology, pathology and psychology. Also methods of opium stimulation, and opium literature, longevity, immaturity and family degeneracy, effects upon age and sex, posology, qualified vincibility, reforms and failures, specific therapeia, therapeutics and hygiene. Narcotic stimuli, alcoholics, opium and alcoholics contrasted. The poppy or the vine. Opium and Cannabis contrasted, tobacco and coca. Coffee and tea in the contrast. Legislation against Stimuli, deductions, conclusions, &c., &c. The book is very instructive, and in all respects highly entertaining. Quotations, statistics, history, literature, &c., &c., constitute the staple composition of the book. The legislation upon the subject of stimulants attract our special attention, the conclusions arrived at are philosophical and logical. Temperance advocates, and general lovers of themselves and of mankind should carefully observe his facts, whether they adopt his conclusions or not, his temperance principles are of high general order, and are based upon an aggregation of facts which fully sustain his conclusions. This book should be placed in the hands of the intelligent and appreciative public as well as the profession. It is published by J. B. Lippincott & Co., Philadelphia.

